# Hospital Accounts

**Published:** 1920-02-21

**Authors:** H. E. Smithers

**Affiliations:** General Manager. 28-29 Southampton Street, Strand, W.C. 2.


### THE UNIFORM SYSTEM OF ACCOUNTS.

The two first following letters appeared in *The Times* of February 14 and 17 respectively.

### HOSPITAL ACCOUNTS.

To the Editor of *The Times*.

Sir,—In the article of Mr. Basil Mayhew in your issue of January 23, page 16, he refers to the "Uniform System of Accounts originated in 1906 by that great hospital accountant, John G. Griffiths." As publishers of "The Uniform System of Accounts for Hospitals and Public Institutions," by Sir Henry Burdett, K.C.B., we desire to point out that the Uniform System of Accounts was originated by Sir Henry Burdett so long ago as 1869, and that this system was subsequently adopted by many British hospitals and institutions. In 1893 was published the first edition of the history, with a description of the system. Several editions followed, the latest being the fourth edition of "The Uniform System of Accounts," with many developments up to date, published by ourselves in 1916.

The Revised Uniform System of Hospital Accounts (as adopted by King Edward's Hospital Fund, the Metropolitan Hospital Sunday Fund, and the Hospital Saturday Fund) is based on the original Uniform System of Sir Henry Burdett, and came into being after an inquiry and report on the subject by Mr. John G. Griffiths, whose report was drawn up "after consultation with numerous hospitals officials and auditors, and with Sir Henry Burdett, to whom the introduction of the original Uniform System had been due." The words in quotation marks are taken from the red book, "The Revised Uniform System of Hospital Accounts," issued in August 1914 by King Edward's Hospital Fund.

Yours faithfully,
For the Scientific Press, Limited,
H. E. Smithers, General Manager.

28-29 Southampton Street, Strand, W.C. 2.

---

To the Editor of *The Times*.

